# Comparative Toxicotranscriptomics of Single Cell RNA-Seq and Conventional RNA-Seq in TCDD-Exposed Testicular Tissue

**DOI:** 10.3389/ftox.2022.821116

**Published:** 2022-05-09

**Authors:** Alex Haimbaugh, Danielle Meyer, Camille Akemann, Katherine Gurdziel, Tracie R. Baker

**Affiliations:** ^1^ Department of Pharmacology, School of Medicine, Wayne State University, Detroit, MI, United States; ^2^ Institute of Environmental Health Sciences, Wayne State University, Detroit, MI, United States; ^3^ Department of Environmental and Global Health, University of Florida, Gainesville, FL, United States; ^4^ Genome Sciences Core, Office of the Vice President for Research, Wayne State University, Detroit, MI, United States

**Keywords:** single cell RNA-seq, TCDD, pseudo bulk-seq, bulk-seq, scRNA-seq dropout, exposure, transcriptome

## Abstract

In this report, we compare the outcomes and limitations of two methods of transcriptomic inquiry on adult zebrafish testes exposed to 2,3,7,8-tetrachlorodibenzo-p-dioxin (TCDD) during sexual differentiation: conventional or bulk RNA-seq (bulk-seq) and single cell RNA sequencing (scRNA-seq) data. scRNA-seq has emerged as a valuable tool for uncovering cell type-specific transcriptome dynamics which exist in heterogeneous tissue. Our lab previously showed the toxicological value of the scRNA-seq pipeline to characterize the sequelae of TCDD exposure in testes, demonstrating that loss of spermatids and spermatozoa, but not other cell types, contributed to the pathology of infertility in adult male zebrafish exposed during sexual differentiation. To investigate the potential for technical artifacts in scRNA-seq such as cell dissociation effects and reduced transcriptome coverage, we compared bulk-sequenced and scRNA-seq-paired samples from control and TCDD-exposed samples to understand what is gained and lost in scRNA-seq vs bulk-seq, both transcriptomically and toxicologically. We hypothesized that the testes may be sensitive to tissue disruption as they contain multiple cell types under constant division and/or maturation, and that TCDD exposure may mediate the extent of sensitivity. Thus, we sought to understand the extent to which this dissociation impacts the toxicological value of data returned from scRNA-seq. We confirm that the required dissociation of individual cells from intact tissue has a significant impact on gene expression, affecting gene pathways with the potential to confound toxicogenomics studies on exposures if findings are not well-controlled and well-situated in context. Additionally, a common scRNA-seq method using cDNA amplified from the 3’ end of mRNA under-detects low-expressing transcripts including transcription factors. We confirm this, and show TCDD-related genes may be overlooked by scRNA-seq, however, this under-detection effect is not mediated by TCDD exposure. Even so, scRNA-seq generally extracted toxicologically relevant information better than the bulk-seq method in the present study. This report aims to inform future experimental design for transcriptomic investigation in the growing field of toxicogenomics by demonstrating the differential information extracted from sequencing cells—despite being from the same tissue and exposure scheme—is influenced by the specific protocol used, with implications for the interpretation of exposure-induced risk.

## Introduction

The transcriptome is the complete picture of all expressed genes in a cell and in what quantity, during a specific developmental stage and/or experimental condition. The field of transcriptomic research has expanded at a rapid rate since the first attempts in the 1990s, gaining granularity and precision with each new advance. What could first only tell us limited transcript abundance in a tissue (SAGE), can now impart entire transcriptomes in high-throughput studies with single-cell resolution. Transcriptome analysis is the most widely used tool in the field of toxicogenomics (TGx) to study gene (dys)regulation in a biological system following chemical exposure (Federico et al., 2020). Due to its importance, there has been a worldwide call for standardization of “omics” data from the TGx community, as guidelines for analysis have not been formally established (Federico et al., 2020). Successful TGx requires best practices in experimental design, data processing techniques, and validation assays in order to produce reliable transcriptomic data which can be efficiently interpreted to serve downstream analyses, such as safety assessments used in regulatory decisions.

With each new method of exploring the gene expression landscape at an increasingly granular level, there are advantages and disadvantages to take into account when planning an experiment. Some methods are more accessible and therefore quite common in labs, such as microarray and RNA-seq. The concept behind microarray is that RNA molecules in a sample can be reversed transcribed to cDNA, and these cDNA sequences can be captured, if present, by oligo probes on the microarray chip. This method certainly advanced the field as microarrays became quite detailed, encompassing entire genomes; gene batteries specifically for toxicology are even available (Lettieri, 2006). RNA sequencing (RNA-seq) uses the same concept of cDNA sequencing as microarray, but allows untargeted exploration of the transcriptomic landscape in a sample of any species for which the genome is annotated. Thus, the advent of RNA-seq revolutionized the field, heralding the next-generation sequencing (NGS) era of “discovery-driven” research. The more complete picture offered by RNA-seq allowed for more in-depth analysis of mechanism of action (MOA), pathway enrichment analysis, and, when combined with phenotypic endpoints, phenotypic anchoring such as biomarkers of disease or prognosis. Together, these valuable insights can inform chemical toxicity and risk assessments. However, despite the broadened opportunities RNA-seq allows, a major limitation to precisely identifying biological relevance with RNA-seq has been the processing of heterogeneous tissues as homogeneous entities, which produces a gene expression signature that is essentially a composite of the responses of the different cell types comprising the tissue. Science is well aware that various cells in a tissue can be highly differentiated and exhibit markedly different expression profiles. Toxicology can benefit from this more precise information as various cell types in diverse tissue can respond quite differently to the same toxicant (Hsu et al., 2020; Wang et al., 2021).

The newer transcriptomic analysis method of single cell RNA-Seq (scRNA-seq) (Tang et al., 2009) mitigates this issue by capturing the complex profiles of the singular cells constituting a tissue. There are various specific scRNA-seq methods, depending on the specific knowledge (Chen et al., 2019), but the overarching goal is acquiring the transcriptome of each cell in a tissue. scRNA-seq builds upon conventional RNA-seq (referred to as bulk-seq; representing the homogeneous, intact state of the tissue) research and is comparable in the basic concept. The sequencing that occurs at the single cell level is essentially the same process cells undergo in bulk-seq; the difference lies in the ability to computationally extricate the results for each cell rather than an averaged output of the entire sample. The advantageous difference of increased granularity is tantamount—scRNA-seq faithfully represents cellular heterogeneity by distinguishing gene expression profiles between different cell types, and even between the same cell type in different cellular states (Lähnemann et al., 2020). The advances in medicine made possible by scRNA-seq are invaluable, e.g., scRNA-seq enabled Zou et al. (2021) to develop a prognostic signature in gastric adenocarcinoma, Deng et al. (2020) identified characteristics predicting CAR T cell therapy efficacy. Toxicology can follow suit to develop targeted therapies for the myriad of multi-cell-type health problems associated with environmental exposure to chemicals, including cancer (Snyder, 2012), asthma (Sachdeva et al., 2019), and infertility (Canipari et al., 2020; Selvaraju et al., 2020).

With this exciting opportunity come inevitable challenges. While scRNA-seq data is structurally similar to bulk-seq data, differences in the tissue- and data preprocessing steps affect replicability, with scRNA-seq more prone to artifacts (e.g., technical or other non-biological sources of variation) (Federico et al., 2020). Contributing to artifacts are tissue dissociation, dropout, and complex quality control measures. The fact that scRNA-seq requires, by definition, single cells that must be individuated from the intact tissue of an organism potentially confounds results. This requirement impacts not only the number of cells available for analysis (those that survive dissociation), but also the physical separation of cells from their network can have an unwanted biological impact on surviving cells. While *in situ* sequencing (the act of sequencing RNA on, for example, a slide of tissue preserved in its natural environment) is possible, the throughput limitations render it undesirable. A more commonly used method for singularizing cells is enzymatic digestion, such as used in the present study. The act of disruption from the native environment and the digestion process (which changes cellular shape among other facets) can potentially result in an altered cellular state, e.g., stress response, and thus findings unrepresentative of the actual cellular mechanisms *in vivo*. However, other methods of tissue dissociation that may prevent such microenvironmental shifts, such as laser capture microscopy and microdissection capillary pipette, are much lower-throughput (Hwang et al., 2018). Dropout events (failure to detect part or all of a transcriptome) and high (Ramsköld et al., 2012; Brennecke et al., 2013; Lun et al., 2016), can permeate due to low capture efficiency, the general stochastic nature of gene expression, and the very low input available (pg-ng) compared to bulk-seq (ng-g). Additionally, workflow pipelines for scRNA-seq and bulk-seq diverge in the complex QC measures required for scRNA-seq data that are not required in bulk-seq analysis. For example, “doublets” and poor quality cells (abnormally low read counts, high mitochondrial mRNA content) must be removed from analysis.

One way to parse artifacts from biological relevance is to validate scRNA-seq data by comparing it to bulk-seq data. In order to make scRNA-seq data comparable to bulk-seq data, a pseudo bulk-seq dataset can be derived from collapsing the reads counts from the various identified clusters (cell types) of scRNA-seq data to mimic *in silico* an average expression profile, such as that produced by bulk-seq on intact tissue (Lähnemann et al., 2020). Theoretically, the results from a pseudo bulk-seq dataset would resemble those from a bulk-seq dataset. However, technical artifacts are introduced by the act of sequencing cells on a microfluidic device rather than at once from a suspension of dissociated cells. A common 3′-end counting droplet-based method of scRNA-seq, 10x Genomics Chromium, is used here. With the expanding field of TGx and increased use of scRNA-seq, we were interested in determining what information was gained and lost compared to the more common, accessible bulk-seq method, in terms of transcriptome coverage, data analysis, and conclusions that can be drawn from both methods.

The field can benefit from seeing these data from different perspectives; apparent inconsistencies are not necessarily misleading and in fact can be quite informative (Qiu, 2020). However, understanding the sources of divergent information and their implications is critical. Our previous toxicological work with scRNA-seq in zebrafish (*Danio rerio*) testes revealed differential cell type population alterations in response to exposure to the classic environmental contaminant 2,3,7,8-tetrachlorodibenzo-p-dioxin (TCDD) (Haimbaugh et al., 2022), which is known to induce male fertility defects in both fish and humans (King Heiden et al., 2009; Baker et al., 2013; Baker et al., 2016; Eskenazi et al., 2018; Bruner-Tran et al., 2019). One marked difference was a loss of spermatids and spermatozoa; in these cells, pathways of apoptosis and sperm disorder were upregulated. We show that these intriguing pathway upregulations resulting from sequencing at the single cell level are not detected with bulk-seq. scRNA-seq detected the change in sperm proportion from about 20% in controls to around 4% in TCDD-exposed cells. In bulk-seq, sperm suffer a similarly reduced physical representation in the sample, but the resulting low signal from these populations may be occluded by competing signals from the overwhelming majority of other cell types. Under bulk-seq conditions, certain aspects of the toxicological profile may be lost to noise, such as toxic effects to relatively few cells. However, we demonstrate that bulk-seq detected immune system modulation that scRNA-seq did not, likely due to cell dropout of immune cells in scRNA-seq. The immune cell population in testes is quite small. We did not detect any immune cells in our scRNA-seq data, and in other scRNA-seq datasets, they comprise just 3% of the overall cell population on average in controls. The required cell dissociation step to perform scRNA-seq can cause cell death, further reducing the number of detected immune cells. scRNA-seq loses about a third of the cell input (10x Genomics Inc., 2021a). Further, by standard bioinformatic processing of scRNA-seq data, cell type populations under 30 cells will not be registered. Minor or unstable cell types such as immune cells may not survive the sample preparation and data processing of scRNA-seq. Early-life exposure to TCDD does not appear to affect adult sperm vulnerability to dissociation, but this potential limitation could apply for other toxicants or other, more acute exposure schemes.

**FIGURE 1:**
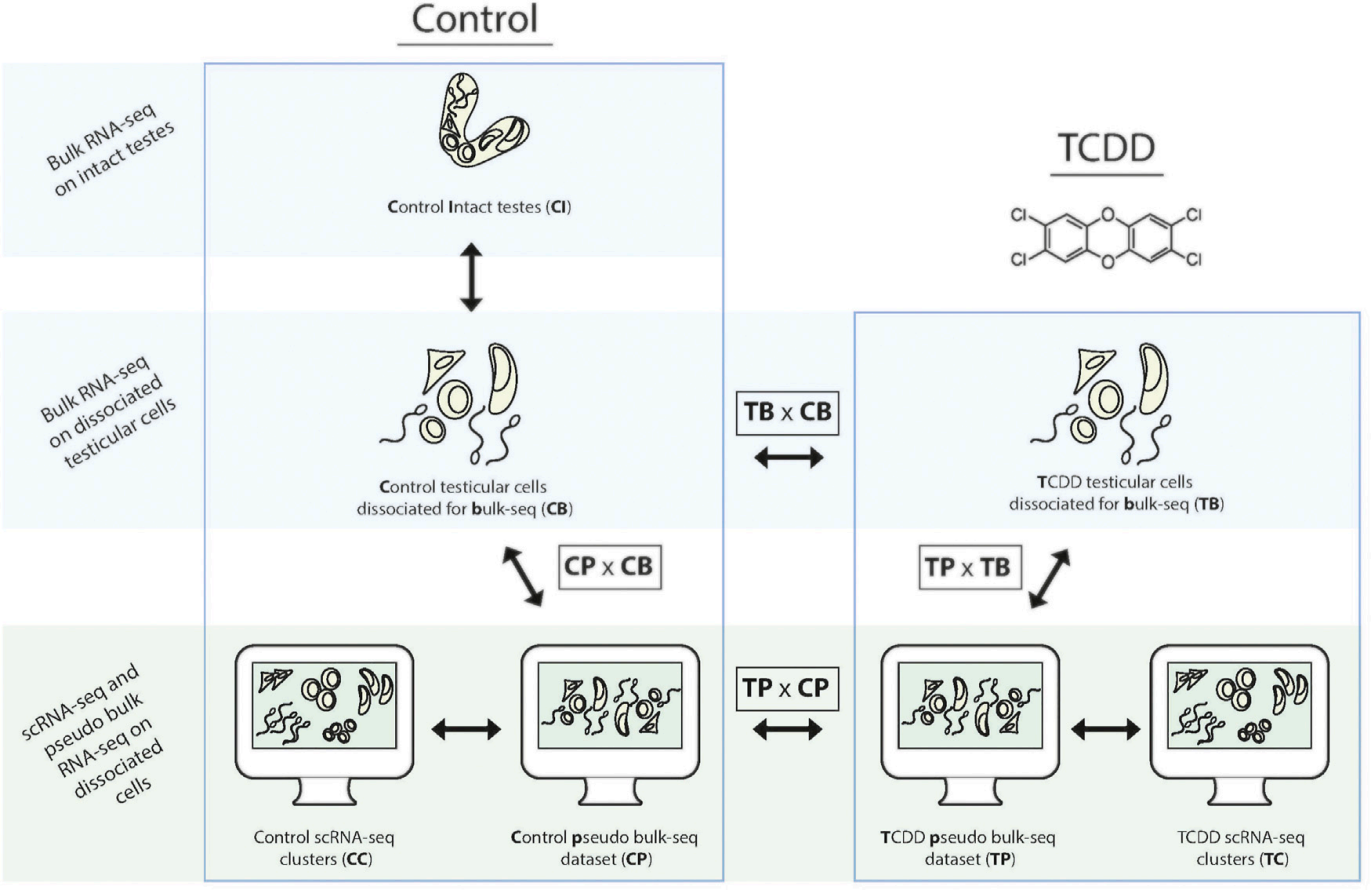
Schematic of the comparative transcriptomic analyses from each method and associated tissue preparation. To determine baseline effects of dissociation, paired samples of intact and dissociated testes were taken for bulk-seq. Bulk-seq of control (CB) and TCDD-exposed dissociated testes (TB) (middle panel) were compared to pseudo bulk-seq of paired dissociated samples that underwent scRNA-seq (CP, TB) (lower panel, middle images). Pseudo bulk-seq samples were compared to their scRNA-seq cluster complement (lower panel, outer images).

In this study we seek to characterize and compare the TGx profile(s) of early-life TCDD exposure, *via* two complementary methods: pseudo bulk-seq of scRNA-seq data, and bulk-seq of dissociated cells. Abbreviations for the various analyses are provided in Figure 1 as reference (i.e., pseudo bulk-seq comparison of TCDD-exposed and control samples is abbreviated TPxCP, while the same comparison in bulk-seq is TBxCB). We first confirm the known fact that indeed, dissociation causes transcriptomic disruption (CDxCI); however, this disruption does not appear to significantly affect TCDD-exposed cells more so than controls (CDxCI vs. TBxCB). Single cell sequencing does have a differential effect on TCDD samples compared to the bulk-seq method (TPxTB vs. CPxCB), despite identical tissue preparation of controls. The toxicotranscriptomic testicular profiles of TCDD exposure delivered by bulk-seq (TBxCB) do differ from that of pseudo bulk-seq (TPxCP), presenting significantly better transcriptome coverage, and especially that of low-expressing transcripts and from cell types of smaller populations; at the same time, giving more global information and potentially less toxicologically relevant information. This report provides perspective to inform methodological design in TGx by demonstrating that differential information extracted from sequencing cells—despite being from the same tissue and exposure scheme—is influenced by the specific protocol used, with implications for the interpretation of exposure-induced risk.

## Results and Discussion

### The Impact of Protocol Choice (Dissociation) on Gene Expression (CDxCI)

**FIGURE 2:**
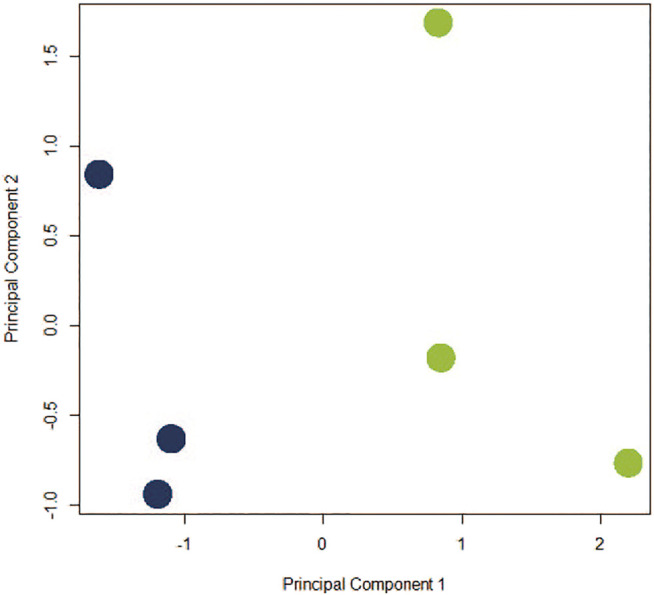
PCA plot depicting co-clustering of gene expression in dissociated replicates (blue) and intact replicates (green). Samples clustered distinctly along PC1 based on method of preparation (negative and positive, respectively).

The assumption in measuring any experimental variable where all other factors (i.e., tissue preparation) are held constant is that any necessary technical manipulation will affect both groups equally and thus avoid confounding the results. Tissue dissociation is expected to produce a broad stress response in cells. Mitochondrial activity (van den Brink et al., 2017), heat shock response (O’Flanagan et al., 2019), cell death (Adam et al., 2017; Wu et al., 2018) and their associated gene expression profiles are known effects of dissociation. Tissue dissociation is a well-established “necessary evil” ingrained in common scRNA-seq protocols. Cells must be individuated for sequencing, yet dissociation of the tissue (whether mechanical, chemical, enzymatic, etc.) induces myriad transcriptional artifacts due to the disrupted cell microenvironment. Machado et al. (2021) found 10–50% of the transcriptome is altered by dissociation alone, and Wu et al. (2018) scRNA-seq data returned a cluster consisting primarily of dissociation-induced artifacts. To characterize the unavoidable impact of dissociation on zebrafish testes, we compared bulk-seq data from enzymatically dissociated testes with intact testes. Following dissociation, 435 differentially expressed genes (DEGs) (*p* < 0.01, LFC ≥1, ≤-1) were significantly upregulated; 348 downregulated. Figure 2 depicts the PCA plot of dissociated and intact samples. Pathway analysis of DEGs confirmed expected differences, such as upregulation of apoptosis, necrosis, and degeneration pathways; downregulation of proliferation and growth pathways (Tables 1, 2).

**TABLE 1:** Ingenuity Pathway Analysis table of disease and Biological Functions from CDxCI DEGs (*p* < 0.01, log fold change >1/<−1). From “Diseases or Functions Annotations'' column, after removing any redundancies, functions without a z-score, or a z-score between −1 and 1. Mapped IDs: 9865; unmapped IDs: 4922.459 analysis-ready molecules: 214 upregulated and 245 downregulated. Ranked by z-score. Left: upregulated pathways. Right: downregulated pathways.

Rank	Upregulated diseases or Functions Annotation	*p*-value	Bias-corrected z-score	Rank	Downregulated diseases or functions annotation	*p*-value	Bias-corrected z-score
1	Necrosis of epithelial tissue	1.15E-06	3.14	48	Cell proliferation of T lymphocytes	0.00119	−1.00
3	Cell death of epithelial cells	1.46E-05	2.99	54	Invasion of tissue	3.61E-08	−1.13
7	Necrosis	8.51E-07	2.79	56	Activation of cells	0.00341	−1.19
9	Apoptosis of epithelial cells	5.81E-06	2.76	73	Growth of genital organ	3.76E-05	−1.65
13	Organ Degeneration	0.00353	2.18	74	Cell movement of epithelial cells	6.98E-05	−1.70
14	Congenital malformation of	6.46E-07	2.05	75	Migration of epithelial cells	9.97E-05	−1.72
15	Dysgenesis	0.000439	1.96	76	Malignant genitourinary solid	0.00415	−1.74
16	Apoptosis	5.94E-10	1.93	79	Growth of organism	4.66E-05	−1.84
22	Aplasia or hypoplasia	0.000818	1.83	80	Proliferation of gonadal cells	7.97E-05	−1.90
30	Oxidation of fatty acid	0.00444	1.62	88	Vasculogenesis	4.54E-07	−2.71
34	Degeneration of testis	0.00283	1.38	—	—	—	—
39	Steroidogenesis of hormone	1.75E-05	1.15	—	—	—	—
40	Congenital malformation of	0.000175	1.14	—	—	—	—
41	Hypoplasia of genital organ	0.000104	1.13	—	—	—	—

**TABLE 2:** Ingenuity Pathway Analysis table of Canonical Pathways from CDxCI DEGs (*p* < 0.01, log fold change >1/<-1). Redundant pathways, pathways with no z-score or z-score between -1 and one removed. Mapped IDs: 9865; unmapped IDs: 4922.459 analysis-ready molecules: 245 upregulated and 214 downregulated. Left: upregulated pathways. Right: Downregulated pathways. Ranked by z-score.

Rank	Upregulated ingenuity canonical pathways	−Log (*p*-value)	z-score	Rank	Downregulated ingenuity canonical pathways	−Log (*p*-value)	z-score
1	Cyclins and Cell Cycle Regulation	2.62	2.65	4	Agrin Interactions at Neuromuscular Junction	4.6	−2.65
2	Aryl Hydrocarbon Receptor Signaling	3.27	1.13	5	ILK Signaling	2.04	−3.16
3	Estrogen-mediated S-phase Entry	6.47	1.13	—	—	—	—

Dissociation is generally expected to affect cell types equally within an experiment, but exposure to environmental contaminants can compromise cell health, rendering exposed cells more vulnerable to threats. In our scRNA-seq data, the contributions of TCDD-exposed sperm population to the overall dataset were dramatically reduced (about 4%) compared to controls (about 23%; a 79% reduction in representation). We previously explored the possibility of this shrinkage being due to the technical requirement of cell dissociation (Haimbaugh et al., 2022). Histology of intact tissue confirmed an apoptosis-driven decrease of 11% in healthy sperm cell representation following exposure, certainly not to the extent observed in scRNA-seq. While apoptosis without dissociation is occurring, we assume the sperm cells that dropped out of scRNA-seq data were toxicologically impaired to the point where dissociation induced cell death in a greater swath of the population. ScRNA-seq has been criticized for its high dropout rate, and we interpret our results as indicating toxicant-related stress could increase the dropout rate in TGx studies.

In bulk-seq exposure data (TBxCB) however, dissociation appears to have minimal impact. The changes evident in TBxCB most likely represent TCDD exposure, and not the tissue preparation method. When examining genes in the Ingenuity Pathway Analysis (IPA) Canonical Pathways of both CDxCI and TBxCB, only three genes are shared of the 113 total genes involved. It is worth mentioning expression of those three overlapping genes (*aldh3b1*, *pklr* and *sat1*), or genes in similar pathways, may be affected by dissociation. An experimental model investigating these genes *via* scRNA-seq may be confounded by dissociation effects. Future scRNA-seq exposure studies should include a dissociation reference in order to parse baseline dissociation effects from toxicologically-relevant changes. In this same vein, “Aryl Hydrocarbon Receptor (AhR) Signaling Pathway” and “Estrogen-mediated S-phase Entry” were upregulated simply by dissociation (CDxCI). AhR and estrogen signaling are a canonical signature of TCDD exposure, and have been thoroughly studied, exclusive of dissociation (Safe et al., 1998; Wilson and Safe, 1998). These findings in context underscore the importance of properly controlling for potential method-specific artifacts in toxicological experiments, which may have to be adjusted for the specific toxicant under study.

### CPxCB: The Droplet-Based Technical Effect

The benefit of scRNA-seq is that population composition can be captured since the transcriptome of each cell in a tissue is represented. At the same time, an established limitation of scRNA-seq is dropout of certain cells and of mRNA detection. Cell dropout can have a physical or technical basis, and transcript dropout has a bioinformatic (QC) basis. The underrepresentation of smaller or rare cell types in scRNA-seq is hypothesized by Denisenko et al. (2019) as being due to differing resistance to cell lysis on the flow cell (thus preempting sequencing) or simply due to irregular cell size or shape preventing entry to the flow cell. Once on the flow cell, scRNA-seq preferentially detects more abundant transcripts, overlooking low-expressing genes (Kharchenko et al., 2014), and has a strong 3′ end bias (Zhang et al., 2019). As for quality control filters, clusters and the cells constituting them are pre-defined by parameters set by the researchers. If these requirements are not met, they are excluded from analysis. The standard definition of a cluster is that it consists of >30 cells. A small cell population’s presence may be grouped into a similar cluster, erasing its unique identity and dropping out of the dataset. A cell is defined as having >500 features (genes) detected. Transcriptome coverage is known to be less extensive in scRNA-seq, with a bias towards detecting longer and more highly expressed transcripts (Macosko et al., 2015; Phipson et al., 2017; Davies et al., 2021). A low-expressing cell or one expressing short transcripts such as transcription factors (TFs), then, could be erroneously removed from analysis as an artifact. Any of these factors could result in artifactual gene expression differences between pseudo bulk-seq and bulk-seq. At least a third of cells are expected to escape capture in the 10x pipeline (10x Genomics, Inc., 2021a). Dropout is assumed proportional across cell types, thus a collapsed scRNA-seq dataset (pseudo bulk-seq) is expected to resemble the bulk-seq dataset from its biological and technical counterpart. Comparing pseudo bulk-seq to bulk-seq data can be used to account for the scRNA-seq cell and transcript dropout rate. The only difference between pseudo bulk-seq on dissociated cells, and bulk-seq on dissociated cells from the same sample, is the technical influence from the introduction of the 10x microfluidic system, where cell lysis and cDNA synthesis occur in oil droplets containing individual cells, rather than as a traditional cell suspension of all cell types at once. We call this technical effect the droplet effect, after the multiple scRNA-seq technologies of encapsulating the cells along with reagents and barcodes in an oil droplet. The assumption is that any differences between the resulting datasets of each method would thus be attributed to the droplet effect. We characterized how the transcriptome profiles of each differ in terms of DEGs and low-expressor coverage.

Of note, the scRNA-seq pipeline appears to have a significant impact on differential gene expression. When comparing control pseudo bulk-seq datasets to control bulk-seq datasets from identical samples, we observed 1,102 significantly upregulated DEGs; 1,029 significantly downregulated. These genes were involved in upregulating pathways of oxidative phosphorylation, cholesterol biosynthesis and cell cycle regulation (Table 3). The most highly upregulated pathway was of oxidative phosphorylation (OXPHOS). One known drawback of scRNA-seq is the cellular stress the required tissue dissociation induces, which is routinely quantified during QC as % mitochondrial content, including OXPHOS genes. The mitochondria release mtRNA under duress, and thus signals an unhealthy, unrepresentative cell. The recommendation is that <10% mitochondrial transcriptional content is required to move forward with a scRNA-seq dataset. Cholesterol biosynthesis can be increased by cell dissociation (Volpe et al., 1985). Both pseudo bulk-seq and bulk-seq samples were dissociated in the same way at the same time, however, there is a short but unquantifiable amount of time between loading cells onto the flow cell and transcriptome capture. Bulk-seq cells were immediately placed in lysis reagent. During this short period between dissociation of both samples and the act of single cell sequencing, there is additional time for these cells to respond to dissociation, which could explain the pathway upregulation in identically-prepared samples. Cell cycle dysregulation is a known, non-biologically relevant source of variation in scRNA-seq data. It is often regressed out of scRNA-seq datasets (Luecken and Theis, 2019; Hérault et al., 2021).

**TABLE 3:** Ingenuity Pathway Analysis table of Canonical Pathways from CPxCB DEGs (*p* < 0.01, log fold change >1/<-1). Redundant pathways, pathways with no z-score or z-score between -1 and one removed. Mapped: 2374; unmapped: 594.1,465 analysis-ready molecules: 735 upregulated and 730 downregulated. Ranked by z-score. Left: upregulated pathways. Right: Downregulated pathways. Ranked by z-score.

Rank	Upregulated ingenuity canonical pathways	-Log (*p*-value)	z-score	Rank	Downregulated ingenuity canonical pathways	-Log (p- value)	z-score
1	Oxidative Phosphorylation	3.62	2.24	34	Coronavirus Pathogenesis Pathway	3.62	−1.06
2	Kinetochore metaphase	4.71	2.06	35	BAG2 Signaling Pathway	2.86	−1.26
3	Assembly of RNA	3.52	1.73	36	Aldosterone Signaling in Epithelial	3.75	−1.26
4	Spliceosomal Cycle	2.34	1.67	37	3-phosphoinositide Biosynthesis	2.03	−1.278
5	Cyclins and Cell Cycle	2.16	1.51	38	Androgen Signaling	2.72	−1.34
6	Superpathway of Cholesterol	2.74	1.41	39	Estrogen Receptor Signaling	5.14	−1.54
7	Pyrimidine	3.8	1.41	40	Cysteine Biosynthesis III	2.23	−1.63
8	Cholesterol Biosynthesis I	2.33	1.34	41	RAN Signaling	2.82	−2.45
9	Cholesterol Biosynthesis II	2.33	1.34	42	Unfolded protein response	2.74	−2.71
10	Cholesterol Biosynthesis III	2.33	1.34	42	Unfolded protein response	2.74	−2.71

As expected, transcriptome coverage was decreased in pseudo bulk-seq, detecting only 6,847 RefSeq mRNA Accession IDs to bulk-seq’s 14,160. We next tested the idea that scRNA-seq data underrepresents shorter and/or less abundant mRNAs. First, we surveyed control transcriptomes for low expressor coverage by defining a low expressing transcript as possessing between one read count and ≤1% of the maximum normalized read count sum within a dataset, and then calculated the percent transcriptome coverage of these low expressors for each method. By setting a filter for bulk-seq data to match the pseudo bulk-seq read count sum lower limit, we investigated how bulk-seq datasets would be hypothetically diminished by the higher read count threshold. The average coverage by bulk-seq then fell by about 30% (*p* = 0.003522, 1-tailed *t*-test) (Table 4). Thus, if the bulk-seq transcript detection sensitivity matched pseudo bulk-seq sensitivity, a sizable population of bulk-seq transcripts would remain undetected. This difference is more important when dealing with only significant DEGs. While the pseudo bulk-seq dataset does not contain low expressors (as defined as 1% of the maximum count sum), about 25% of low expressors are significant DEGs in the bulk-seq dataset. When the filter was applied to match the pseudo bulk-seq sensitivity, this dropped to around 7% (*p* = 0.045294, 1-tailed *t*-test). The presumed absence of these genes in single cell datasets could affect interpretation of the results.

**TABLE 4:** Coverage of low expressors is reduced in bulk-seq when scRNA-seq filters apply. Low expressor reads counts are an average across all control samples (CD, CI, CB).

		Detected transcripts	Detected transcripts (significant DEGs)
No filter	Low expressor reads count range	1–823,127	1–178,033
	Low expressor coverage	51.81%	24.45%
Filter	Low expressor reads count range	106–823,127	106–178,033
	Low expressor coverage	36.62%	7.20%
—	*p*-value	0.003522	0.045294

Second, using transcription factors (TFs) as a proxy for both short and low-expressing transcripts, we searched the pseudo bulk-seq and bulk-seq dataset for the available list of 3,068 *Danio rerio* TFs (ATFDBv3.0) (AnimalTFDB3, 2021). Fewer TFs were detected in the pseudo bulk-seq dataset compared to bulk-seq, and this slight trend is exaggerated for significant DEGs (Table 5). Further, none of these significant TFs are shared between the two methods. In fact, the entire population of TFs detected by pseudo bulk-seq is a significantly different population than the significant bulk-seq DEGs (*p* = 2.175e-05, chi-squared test), with only seven genes overlapping. Thus, a study using solely one method would receive a one-dimensional representation of the data, and may not cover TFs of interest. “Missing” genes in either dataset, by virtue of their absence, could influence the toxicological interpretation of experimental findings.

**TABLE 5:** Detection of transcription factors is reduced in pseudo bulk-seq. TBxCB: TCDD bulk-seq vs control bulk-seq. TPxCP: TCDD pseudo bulk-seq vs control pseudo bulk-seq. Cells pertaining to significant DEGs are shaded. Chi-squared test.

	Detected transcripts	Detected transcription factors	Detected transcripts (significant DEGs)	Detected transcription factors (significant DEGs)
TBxCB	14,160	1,393 (9.8%)	310	63 (20.3%)
TPxCP	6,847	487 (7.1%)	1,099	44 (4%)
*p*-value	NA	6.84e-10	NA	3.14e-21

**FIGURE 3:**
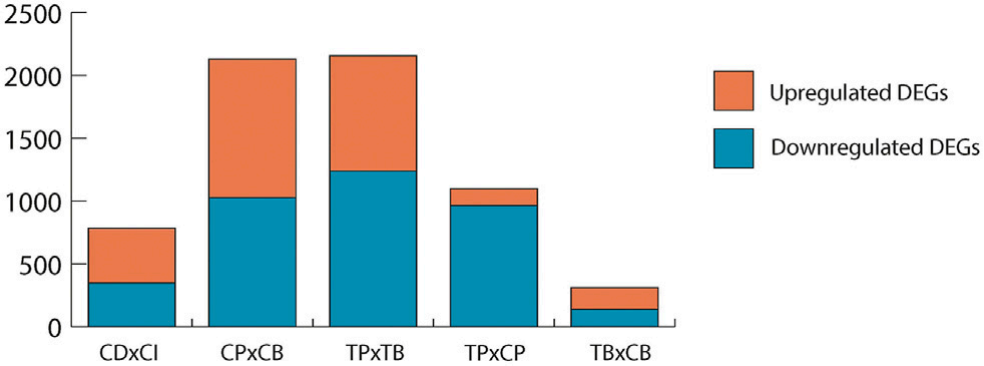
Significant DEGs (*p* < 0.01, LFC ≥1 or ≤ −1) in each comparison. Upregulated: LFC ≥1; downregulated: LFC ≤ −1. CDxCI: paired control samples of dissociated and intact testes used for bulk-seq. CPxCB: paired control samples of dissociated testes used for pseudo bulk-seq and bulk-seq. TPxTB: paired TCDD-exposed samples of dissociated testes used for pseudo bulk-seq and bulk-seq. TPxCP: pseudo bulk-seq analysis of paired dissociated control and TCDD-exposed testes. TBxCB: bulk-seq analysis of paired dissociated control and TCDD-exposed testes.

While scRNA-seq pipleine itself is not assumed to affect unexposed, healthy cells any differently than cells exposed to environmental contaminants, it is important to determine if the established baseline changes in DEGs, gene ontology, and low expressor coverage resulting from the droplet effect in controls (CPxCB) would remain in TCDD-exposed samples (TPxTB). If TCDD exposure exerted no influence on scRNA-seq processing, a high overlap would be expected among each aspect of CPxCB and TPxTB. The number and fold change direction of significant DEGs held steady in TPxTB comparisons (1,239 up-; 917 downregulated) as compared to CPxCB (Figure 3), with considerable overlap (about 60%).

The core pathways associated with these DEGs (Table 6) were also similar to pathways of CPxCB. About 40% of CPxCB and TPxTB pathways overlap, including oxidative phosphorylation, cholesterol biosynthesis, and cell cycle regulation upregulation. These overlapping main pathways demonstrate the general influence of the droplet effect on gene expression from otherwise identically prepared samples and are not toxicologically relevant to TCDD exposure. In fact, cholesterol biosynthesis has been thoroughly shown to decrease following TCDD exposure (Fletcher et al., 2005; Sato et al., 2008; Tanos et al., 2012; Fader et al., 2017). However, it is important to note that 60% of pathways were unique to TCDD; other chemicals may have more or less of an effect. Bulk-seq detection of low-expressing transcripts and TFs is not affected by TCDD exposure, before or after filters were applied to match pseudo bulk-seq coverage. Coverage reduction is simply a result of the pseudo bulk-seq method and is not mediated by exposure. By these three measures, TCDD exposed testes cells do not appear differentially susceptible to the technical effects of the scRNA-seq pipeline.

**TABLE 6:** Ingenuity Pathway Analysis table of Canonical Pathways from TPxTB DEGs (*p* < 0.01, log fold change >1/<-1). Redundant pathways, pathways with no z-score or z-score between -1 and one removed. Mapped IDs: 3550; unmapped IDs: 838.184 analysis-ready molecules: 103 upregulated and 81 downregulated. Ranked by z-score. Left: upregulated pathways. Right: Downregulated pathways.

Rank	Upregulated ingenuity canonical pathways	z-score	Rank	Downregulated ingenuity canonical pathways	z-score
1	Oxidative Phosphorylation	2.52	12	p70S6K Signaling	−1.07
2	Cholesterol Biosynthesis I	2	13	mTOR Signaling	−1.07
3	Cholesterol Biosynthesis II (*via* 24,25-dihydrolanosterol)	2	14	Insulin Receptor Signaling	−1.21
4	Cholesterol Biosynthesis III (*via* Desmosterol)	2	15	Remodeling of Epithelial Adherens Junctions	−1.34
5	Superpathway of Cholesterol Biosynthesis	1.89	16	Gluconeogenesis I	−1.41
6	Cell Cycle Control of Chromosomal Replication	1.60	17	D-myo-inositol-5-phosphate Metabolism	−1.41
7	Assembly of RNA polymerase II Complex	1.07	18	HIF1α Signaling	−1.53
—	—	—	19	Glycolysis I	−1.63
—	—	—	21	Sirtuin Signaling Pathway	−1.71
—	—	—	22	3-phosphoinositide Biosynthesis	−1.79
—	—	—	23	Superpathway of Inositol Phosphate Compounds	−1.96
—	—	—	24	Androgen Signaling	−2
—	—	—	25	Coronavirus Pathogenesis Pathway	−2.04
—	—	—	26	Estrogen Receptor Signaling	−2.14
—	—	—	27	Autophagy	−2.35
—	—	—	28	IGF-1 Signaling	−2.50

### Clustering Reveals Differential Susceptibility to scRNA-Seq Dropout (TPxCP)

Despite no differences in technical dropout between exposed cells and control cells, clustering of the TCDD sample by scRNA-seq revealed near-total physical dropout of spermatids and sperm, beyond what is attributed to exposure. We have shown in intact tissue that a significant percent of sperm and spermatid populations apoptose in response to TCDD exposure (Haimbaugh et al., 2022), but the remaining healthy sperm and spermatids contribute similarly to the overall population as control sperm and spermatids. In scRNA-seq clusters, these two populations are diminished by about 80%. Other cell types are not affected as drastically, in fact, the proportion of spermatogonial stem cells did not change. Dropout in scRNA-seq is assumed to be distributed proportionally across all cell types in a sample. However, in testes cells with a history of TCDD exposure, dropout is differentially experienced by late germ cells. These late germ cells are known to be under strain, as many are undergoing apoptosis. The stress background of a tissue may influence cell dropout in the scRNA-seq pipeline as the added mitochondrial duress and increased latency to lysis on the flow cell could be an insurmountable affront to already-unstable cells from both exposure and the combination of exposure and dissociation mentioned above.

### Toxicotranscriptomic Profile Representation in Bulk-Seq vs. Psuedo Bulk-Seq (TBxCB vs. TPxCP)

**FIGURE 4:**
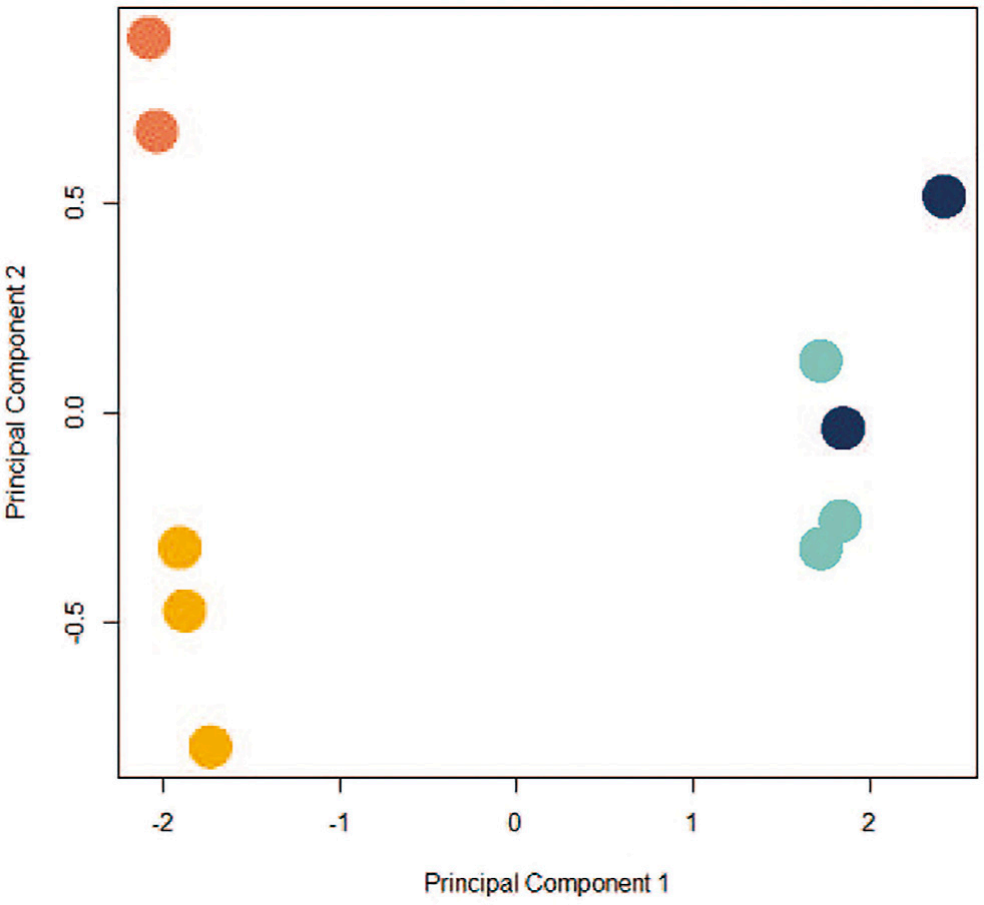
PCA plot depicting co-clustering in CBxTB and CPxTP. Control bulk-seq (CB) replicates: light orange; TCDD-exposed bulk-seq (TB) replicates: dark orange; control pseudo bulk-seq (CP) replicates: light blue; TCDD-exposed pseudo bulk-seq (TP) replicates: dark blue. Samples clustered distinctly along PC1 based on method of preparation (bulk-seq (CB, TB) or pseudo bulk-seq (CP, TP); negative and positive, respectively), independently of exposure status. Control and exposed replicates from either preparation clustered along PC2 (exposed replicates tended to cluster above the control replicates).

Given the ways dissociation and individual sequencing can affect control and exposed cells (CPxCB, TPxTB), and the growing interest in using scRNA-seq for TGx studies, we next examined the toxicologically-relevant differences in profiles between bulk-seq (TBxCB) and pseudo bulk-seq (TPxCP) exposure datasets. Since pseudo bulk-seq is a collapsed transcriptome of every individual cell, it is expected to resemble the bulk-seq averaged transcriptome. Any differences in the two datasets can help estimate the cell populations undergoing dropout in scRNA-seq. If the pattern of cell dropout does not meet the expectation that all cell types will be affected equally, this suggests the sequencing process itself contributed to the sacrifice of a cell population. In the bulk-seq comparison, 310 genes were significantly differentially expressed (171 upregulated and 139 down-; *p* < 0.01; LFC ≥1 or ≤ −1) between TCDD-exposed and control; the pseudo bulk-seq comparison contained 1,099 significant DEGs (134 up- and 965 down-). The greater number of DEGs in scRNA-seq is likely a product of noise due to both cell and transcript dropout. Disproportionate cell-type dropout in scRNA-seq TCDD samples produces a composition of cells unlike that remaining in the bulk-seq TCDD samples, where dropout does not apply. The increased incomparability of cell type populations embeds noise in the system. Transcript dropout from lower transcriptome coverage in scRNA-seq results in a decreased signal:noise ratio. Figure 4 shows the differences between the control and TCDD samples for each method.

**FIGURE 5:**
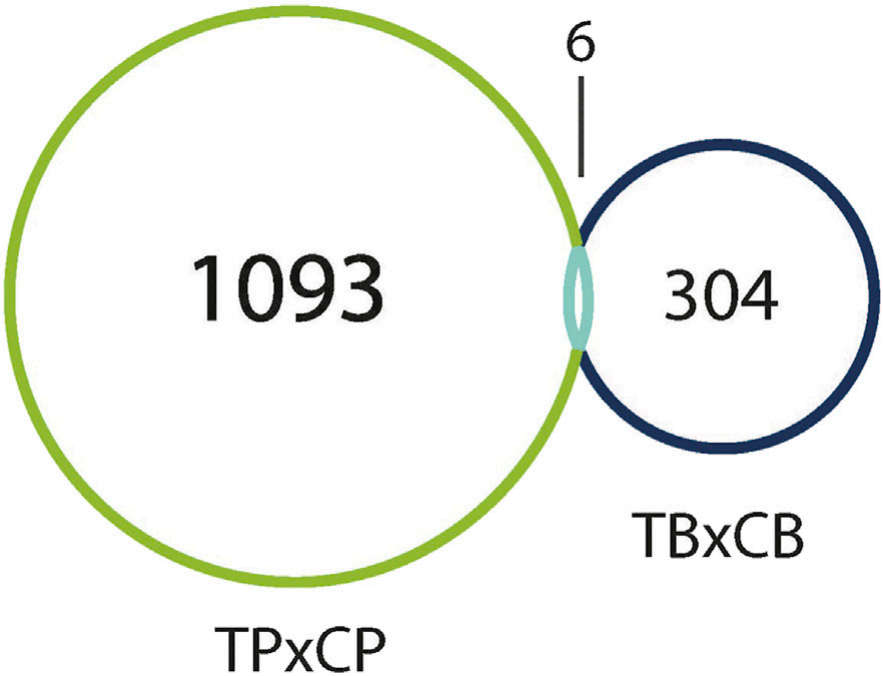
Venn diagram of DEGs (both up- and downregulated) in TCDD-exposed pseudo bulk-seq preparation (left), and bulk-seq preparation (right) as compared to control. Pseudo bulk-seq detected 1,093 DEGs that were undetected in bulk-seq. Conversely, 304 genes were detected using bulk-seq, but not pseudo bulk-seq. 6 DEGs were detected by both methods.

Interestingly, only six of these DEGs overlapped between the two preparations (Figure 5). A common use of scRNA-seq is identification of cell-type markers of interest for further experimentation. Populations expressing a particular marker are then isolated from new tissue, and bulk-seq is performed to allow for deeper sequencing coverage. The markedly different DEG profiles between pseudo bulk-seq and bulk-seq could mislead marker identification and subsequent experiments. Despite low overlap in DEGs, it is possible the transcriptomic profiles of each method converged on toxicologically-relevant pathways, therefore, we compared the overarching functions represented by each method (Tables 7, 8). The 43 pathways generated from pseudo bulk-seq data seem to convey more specific toxicological functions (teratozoospermia, impaired cilia formation), and it is clear the reproductive system has been affected. With the 55 bulk-seq pathways, it is clear basic cellular functions are under distress (ion homeostasis, apoptosis, ROS production), but without prior knowledge, it would be difficult to assume the tissue in question, as they range over less informative pathways. Additionally, the pseudo bulk-seq results of sperm disorder, oligozoospermia, etc., reflect the phenotypic infertility and the lowered male-mediated fertilization rates we have observed following TCDD exposure. There were three exact overlapping pathway annotations from pseudo bulk-seq and bulk-seq including cancer of secretory structure, and two non-specific cancer pathways. The prominence of sperm-related pathways in pseudo bulk-seq data and absence of such in the bulk-seq pathway list may be explained by the observed dropout of sperm cells in scRNA-seq. The sperm-specific signal may have been overridden by the abundance of other cell-type signals in bulk-seq, such as from spermatocytes (45% of all cells, Haimbaugh et al., 2022). In fact, meiosis-related pathways are well-represented in the pseudo bulk-seq results but absent from bulk-seq. This lack of focus could also be due to the relatively low number of IPA-ready molecules for bulk-seq (206) compared to pseudo bulk-seq (696). The full list of IPA results is Supplemental File S1.

**TABLE 7:** Ingenuity Pathway Analysis table of disease and Biological Functions from TPxCP DEGs (*p* < 0.01, log fold change >1/<−1). From “Diseases or Functions Annotation'' column, after removing any redundancies, functions without a z-score, or a z-score between −1 and 1. Mapped IDs: 4,926; unmapped IDs: 1,921 (raw data), after settings of logFC >1 or < −1/*p* < 0.01.696 analysis-ready molecules: 89 upregulated and 607 downregulated. Ranked by z-score. Left: upregulated pathways. Right: downregulated pathways.

Rank	Upregulated diseases or functions annotation	*p*-value	Activation z-score	Rank	Downregulated diseases or functions annotation	*p*-value	Activation z-score
1	Sperm disorder	2.63E-05	3.45	67	Ploidy of cells	1.07E-05	−1.08
2	Carcinoma	2.39E-31	3.30	78	Recombination	1.78E-06	−1.15
7	Nonhematologic malignant neoplasm	1.74E-34	2.79	79	DNA recombination	1.78E-06	−1.15
8	Extracranial solid tumor	3.88E-31	2.74	80	Recombination of cells	6.22E-05	−1.22
9	Tumorigenesis of epithelial neoplasm	0.000777	2.71	81	Cell movement of sperm	2.07E-08	−1.34
10	Non-melanoma solid tumor	9.45E-31	2.68	83	Cycling of centrosome	0.000515	−1.45
11	Teratozoospermia	2.81E-05	2.65	84	Formation of cilia	1.12E-26	−1.47
14	Genitourinary tumor	0.000236	2.51	85	Homologous recombination	6.43E-05	−1.53
15	Genitourinary carcinoma	6.42E-05	2.43	87	Meiosis of germ cells	2.81E-05	−1.72
18	Laterality defect	6.43E-08	2.42	88	Smoothened signaling pathway	3.83E-12	−1.73
19	Malignant genitourinary solid tumor	0.00013	2.38	89	Cell surface receptor linked signal transduction	2.72E-05	−1.73
20	Heterotaxy or ciliopathy	1.31E-33	2.24	90	Formation of cellular protrusions	1.88E-06	−1.76
22	Regional congenital anomaly	4.73E-07	2.22	91	Organization of cytoplasm	1.09E-07	−2.19
24	Oligozoospermia	0.000117	2.14	92	Organization of cytoskeleton	4.15E-06	−2.19
25	Solid tumor	4.03E-31	2.09	93	Microtubule dynamics	5.28E-06	−2.18

**TABLE 8:** Ingenuity Pathway Analysis table of disease or Functions from TBxCB DEGs (*p* < 0.01, log fold change >1/<−1). From “Diseases or Functions Annotations” column, after removing any redundancies, functions without a z-score, or a z-score between −1 and 1. Mapped IDs: 9,868/unmapped IDs: 4,924 (raw data), after settings of log fold change >1 or < −1, *p* < 0.01.206 analysis-ready molecules: 114 upregulated and 92 downregulated. Ranked by z-score. Left: upregulated pathways. Right: downregulated pathways.

Rank	Upregulated diseases or functions annotation	*p*-value	Activation z-score	Rank	Downregulated diseases or functions annotation	*p*-value	Activation z-score
1	Neurotransmission	2.66E-07	2.53	41	Ductal carcinoma	0.00093	−1.67
2	Transport of cation	0.00014	2.20	42	Extraintestinal functional disorder	8.1E-06	−1.74
3	Transport of ion	1.57E-05	2.07	43	Morbidity or mortality	1.52E-05	−1.79
4	Transport of inorganic cation	0.000174	1.96	44	Migration of endothelial cells	0.000904	−1.90
5	Transport of metal ion	0.000314	1.96	45	Cell movement of antigen presenting cells	0.000257	−2.15
6	Transport of molecule	1.77E-06	1.77	46	Cell movement of macrophages	0.000878	−2.56
7	Ion homeostasis of cells	3.39E-06	1.63	47	Cell movement of phagocytes	0.000715	−2.58
8	Growth of organism	0.000509	1.61	48	Cellular infiltration by phagocytes	0.000381	−2.63
9	Colon tumor	0.000743	1.43	49	Cellular infiltration by leukocytes	0.00106	−2.73
10	Transport of metal	0.000106	1.43	50	Cellular infiltration by myeloid cells	0.000528	−2.76
11	Depolarization of cells	9.93E-05	1.39	51	Cell movement of leukocytes	0.000371	−2.76
12	Synthesis of reactive oxygen species	0.000636	1.34	52	Leukocyte migration	0.000582	−2.76
13	Incidence of tumor	7.45E-10	1.32	53	Cellular infiltration by blood cells	0.000574	−2.85
14	Apoptosis	5.7E-10	1.27	54	Cellular infiltration	0.000735	−3.01
15	Genital tumor	7.13E-07	1.27	55	Cell movement of myeloid cells	0.00102	−3.15

Bulk-seq pathways included a potential aspect of the exposure that the pseudo bulk-seq dataset did not: immune system damage or suppression. The most downregulated pathways in bulk-seq are immune-related, while in scRNA-seq, there is no indication of changes in immune function. TCDD is a known immunotoxicant (Warren, 2000; Marshall and Kerkvliet, 2010). Immune cells, while serving important functions in the testes, are such a small population they are often not detected in scRNA-seq experiments, or at very low representation averaging about 3% (Green et al., 2018; Guo et al., 2018; Wang et al., 2018; Jung et al., 2019; Sohni et al., 2019; Shami et al., 2020; Yang et al., 2020). We did not detect immune clusters in our scRNA-seq dataset. It follows, then, that without enriching a sample for immune cells prior to scRNA-seq, immune system information may be lost. The revelation of this loss between our bulk and single cell data reiterates the importance of hypothesis-driven research. ScRNA-seq in general has been rightly touted as a useful tool in discovery-based research which can be mined for relevant information. However, with the testes it may be the case that when studying environmental contaminants known or suspected to be immunotoxic, the *a priori* decision to enrich for immune cells in a scRNA-seq study would produce a more realistic and meaningful dataset.

### scRNA-Seq Clusters Deliver Crucial Toxicological Details (TCxCC)

As we have seen with the comparison of bulk-seq (TBxCB) to pseudo bulk-seq (TPxCP), the narrowing of focus from a broad-ranging question of global expression, to a more specific inquiry that takes into account heterogeneity of a tissue, can impact the biological or toxicological understanding of the results. The same appreciation of granularity applies when comparing a pseudo bulk-seq dataset (TPxCP) to its scRNA-seq cluster counterparts (TCxCC). Clusters are distinct entities providing unique information about cell types in a tissue, and are the main deliverable of scRNA-seq. Two indispensable advantages of clustering are receiving the both proportions of cell type (or cellular state) constituting a tissue, and the gene expression signature of each cluster. Pseudo bulk-seq is not meant to provide that information. Pseudo bulk-seq data collapses scRNA-seq datasets to obtain a broad overview of those extricated clusters. Collapsing the transcriptomic dimensionality from individual clusters into one generalized dataset is also useful in checking for dropout as described above, but will naturally fail to retain all cell-specific changes. This loss of specificity is greater when comparing clusters to bulk-seq data, despite bulk-seq containing the same cell types. This bulk-to-cluster comparison is still important, however, to estimate dropout, as discussed above. Here we compare representations of the transcriptome from scRNA-seq clusters to pseudo bulk-seq and to bulk-seq data following TCDD exposure.

In previous work, we captured ten scRNA-seq clusters spanning all testicular cell types in control fish testes (spermatogonial stem cells (SSC), spermatogonia (SPG), four stages of spermatocytes (SPC), round and elongating spermatids, and two sperm clusters) (Haimbaugh et al., 2022). In TCDD-exposed fish testes, only eight clusters remained--sperm and late spermatid populations were decimated. A total of 980 genes were significantly differentially expressed among each of the eight control and TCDD clusters. 574 of these DEGs were not included in pseudo bulk-seq DEGs; only 243 DEGs overlapped. These differing representations result from the collapse of transcriptomic information across all cells in pseudo bulk-seq. Spermatogenesis is a multi-step process, where transcriptional programs that are turned on in earlier stages must be silenced in later stages as the cell’s needs fluctuate. Each subsequent developmental stage in the male germ cell trajectory is quite different from the one preceding it. In SSCs, self-renewal and mitosis occur. Some of those progeny will remain SSCs, and some will differentiate to SPG. SPG proliferate and then meiose into SPCs, which undergo a second meiosis to form round spermatids. These round spermatids undergo dramatic architectural elongation and compaction to produce the mature spermatozoa. This can lead to the effect of some transcript expression levels “cancelling out,” as one subpopulation expresses, for example, a mitosis program where DNA must be accessible for synthesis, while another compacts chromatin quite tightly to shape sperm.

This divergent transcriptional representation in clusters is exaggerated with bulk-seq. Bulk-seq, being an average expression profile of all cells in the suspension, has the same issues as pseudo bulk-seq would in terms of transcriptional programs “cancelling out,” but with the added noise of the dropout manifesting in the clusters. The uneven dropout in scRNA-seq results in a different sample composition than that of bulk-seq, as described above. Here, 800 DEGs were unique to clusters, with only 17 DEGs overlapping with bulk-seq DEGs.

Without the cluster information, it would’ve been difficult to predict the widespread apoptosis from bulk-seq or pseudo bulk-seq alone, as pathway analysis returned apoptosis pathways with either weak (<1) z-scores, or only one apoptosis result of 55 total pathways (1.8%), respectively. From the clusters we could determine the cell population proportions changed from sperm and spermatids contributing about 30% of the control population, to about 4% after exposure. As a result of the population shift, SPG and early SPC made up about 80% of the TCDD population, whereas in controls they constituted about 30%. From this population shift we were able to hypothesize either a failure in spermiogenesis, or cell death of sperm. Cell death of fully developed sperm was confirmed by immunohistochemistry (Haimbaugh et al., 2022).

## Summary

Acceleration in the field of transcriptomics has brought about myriad useful, high-powered technologies for investigating every aspect of gene expression. Deliberately choosing the method to best understand the specific research question becomes complicated, yet remains critical. This is pertinent for any discipline, including the growing field of TGx. The implications for regulatory toxicology cast a layer of added urgency to this task. We demonstrate here the nuances associated with two common transcriptomic assays (bulk-seq and scRNA-seq), using an early-life TCDD exposure model. Due to cell and transcript dropout combined with reduced transcriptome coverage in scRNA-seq, these two assays offer incomparable DEG profiles. As scRNA-seq is often used for cell-type marker discovery for future deeper sequencing with bulk-seq, the opposing DEG profiles could complicate marker enrichment and subsequent interpretation of the results. Despite originating from the same tissue and same exposure, the pathways these DEGs contribute to were also divergent, with scRNA-seq pathways offering insight into biological mechanisms of sperm loss following TCDD, while bulk-seq presented a profile of general immune dysregulation. Both pieces of evidence, however, are true. These different perspectives reinforce the need for validation efforts using other methods including phenotypes, histology, and behavior analysis, to supplement transcriptomic findings from different analytical tools.

There are efforts to make bulk-seq and scRNA-seq more cohesive. Spatial transcriptomics, while suffering from low throughput, addresses the question of dropout: no dissociation, microfluidic droplet or complex data analysis are required (10x Genomics, Inc., 2021b). Deconvolution techniques are actively being developed, where scRNA-seq data is used to estimate proportions of cell type populations in homogeneous bulk-seq samples (Hunt et al., 2018; Newman et al., 2019; Wang et al., 2019). A potential limitation to this approach is that all cell populations may not be present in the scRNA-seq matrix; we have shown this in testes. The ability to extract information from bulk-seq with scRNA-seq-level specificity would preempt scRNA-seq-specific artifacts and reduce experimental costs dramatically.

Exposure-induced risk is notoriously difficult to determine. The “exposome,” or the multiple exposures each individual ever encounters over their lifetime (Wild, 2005), to-date represents an epistemic limit of toxicology, and a profound caveat in epidemiological studies. Total environmental control can be accomplished only in model organisms. Even with this level of control, the toxicotranscriptomic outcomes of exposure can be differentially represented according to the investigational protocol. With this evident variability, it is critical for researchers to produce reliable, replicable data for toxicological risk interpretation in humans. Zebrafish in particular are uniquely positioned to investigate questions of both aquatic and human toxicology, due to their high potential for translatability. We show the transcriptome of adult zebrafish testes is susceptible to early-life TCDD exposure, which can differentially present as sperm death or immunotoxicity, depending on the assay and its associated strengths and artifacts. Future toxicotranscriptomic studies in other tissues, species, toxicants, or exposure schemes may also find differential results between bulk-seq and scRNA-seq. These differential results are not necessarily misleading, and can in fact enhance the TGx field’s understanding of the cellular and transcriptional states complexly affected by exposure.

## Methods

### Fish Husbandry

Zebrafish (AB strain) were maintained as described in Meyer et al. (2018). Briefly, fish were fed twice daily and kept on a 14:10 h light/dark cycle (Westerfield, 2000) in buffered, recirculating, reverse osmosis water with temperatures maintained at 27–30 °C. Animal use protocols were approved by the Institutional Animal Care and Use Committees at Wayne State University and the University of Wisconsin-Madison, according to the National Institutes of Health Guide to the Care and Use of Laboratory Animals (Protocol No. M00489).

### TCDD Exposure

TCDD (>99% purity) (Chemsyn, Concord, ON, Canada) was used as a 0.4 ng/ml stock solution in dimethyl sulfoxide (DMSO) Zebrafish were exposed as previously described (Henry et al., 1997; Baker et al., 2013). Briefly, fish were exposed at three wpf and again at seven wpf to water-borne TCDD (50 pg/ml) or vehicle (0.1% DMSO) for 1 h each time in small glass beakers with gentle rocking. Fish were raised in beakers with daily water changes of 40–60% at a density of five fish per 400 ml beaker between 3 and 6 weeks, and five fish per 800 ml beaker between 6 and 9 weeks post-fertilization. All results are derived from three independent TCDD exposure experiments done in successive blocks.

### Testes Isolation: Intact Control Testes (CI)

Adult (1-year-old (+/- 1 month)) male zebrafish were euthanized in tricaine methanesulfonate (1.67 mg/ml) (Fisher Scientific, Waltham, MA, United States) for 10 minutes. Testes were dissected and excess adipose tissue removed in ice cold 1x PBS (Gibco, Waltham, MA, United States). Testes were placed in 300 μL RNALater (Thermo Fisher, Waltham, MA, United States) for 48 h. RNALater was then drained and tissue stored at -80 °C.

### Testes Isolation and Enzymatic Dissociation of Testes: Control Dissociated (CD); TCDD and Control Dissociated for 10x Sequencing (TP, CP); TCDD and Control Dissociated for Bulk Sequencing (TB, CB)

Adult (1.5-year-old (+/- 1 month) (TP, CP, TB, CB) or 1-year-old (+/- 1 month) (CD)) male zebrafish were euthanized in tricaine methanesulfonate (1.67 mg/ml) (Fisher Scientific, Waltham, MA) for 10 minutes. For CD replicates (n = 3), only one testis was dissected for dissociation, while the contralateral testis remained intact for paired bulk-seq. For all other replicates, both testes were dissected (CB/CP: n = 3; TB/TP: n = 2). Testes were dissected and excess adipose tissue removed in ice cold 1x PBS (Gibco, Waltham, MA, United States). Testes were minced, then centrifuged for 5 min at 500 g. PBS was removed, and 100 uL of digestion media (100 uL Leibovitz’s L-15 medium (MilliporeSigma, Burlington, MA, United States), one uL bovine serum albumin (New England BioLabs, Ipswich, MA, United States), one uL DNAseI (Zymo Research, Irvine, CA, United States), and 1 mg collagenase Type II (Worthington Biochemical Corporation, Lakewood, NJ, United States)) was added. Tissue was shaken at 280 rpm for 1.5 h, with manual disruption *via* wide-bore pipetting every 15 min. Cells were centrifuged for 5 min at 500 g, digestion media aspirated, and cells resuspended in PBS. Dead cells were removed with a Dead Cell Removal Kit (Miltenyi Biotec, Bergisch Gladbach, Germany). Cell viability of 90% was determined using the BioVision Live/Dead Cell Viability Assay Kit (BioVision Inc., Milpitas, CA, United States), according to manufacturer’s instructions. Cells were immediately 1) loaded for 10x sequencing (CP (n = 3), TP (n = 2)), or 2) placed in Qiazol (Qiagen, Hilden, Germany) and frozen at −80°C (CB (n = 3), TB (n = 2)). Approximately 5,000 cells were loaded for scRNA-seq per replicate (CP, TP). The remaining cells (approximately two million) from the testes suspension were reserved for bulk seq (CB, TB). All cells (approximately one million) from each CD testis (n = 3) were used for bulk-seq,

### RNA Isolation

RNA was isolated from testes using the RNeasy Lipid Tissue Mini Kit (Qiagen, Hilden, Germany) according to the manufacturer’s specifications. Briefly, samples were homogenized in Qiazol (Qiagen, Hilden, Germany), RNA was separated from organic material with chloroform-isoamyl alcohol mixture (≥99.5%) (Millipore Sigma, Burlington, MA, United States), RNA was purified on a filter and eluted with RNAse-free water. RNA concentration was measured with Qubit 3.0 Fluorometer (Invitrogen, Carlsbad, CA, United States). Isolated RNA was stored at -80 °C.

### 3′-End Library Preparation, Sequencing, and Alignment

3′ mRNA-seq libraries were prepared from isolated RNA using QuantSeq 3’ mRNA-Seq Library Prep Kit FWD for Illumina (Lexogen, Vienna, Austria). Samples were normalized to 40 ng/μL (total input of 200 ng in 5 µL) and amplified at 17 cycles. Libraries were quantified using a Qubit 3.0 Fluorometer and Qubit^®^ dsDNA Broad Range Assay Kit (Invitrogen, Carlsbad, CA, United States), and run on an Agilent TapeStation 2200 (Agilent Technologies, Santa Clara, CA, United States) for quality control. The samples were sequenced on a HiSeq 2500 (Illumina, San Diego, CA, United States) in rapid mode (single-end 50 bp reads). Reads were aligned to *D. rerio* (genome assembly GRCz11 (danRer11)) using the BlueBee Genomics Platform (BlueBee, Rijswijk, Netherlands). Raw data and processed files were uploaded to the NCBI GEO database (GSE193758).

### 10x Library Preparation and Sequencing

Single cell transcriptome profiles were generated using the 10x Chromium Controller v2 chemistry following the Chromium Single Cell 3′ protocol. We acquired 180 million reads per sample, or ∼120,000 reads/cell. Raw data and processed files were uploaded to the NCBI GEO database (awaiting approval).

### 10x Data Processing

Cell Ranger was used to align sequencing reads to the zebrafish reference genome (dR10) which was constructed using the mkref command (Zheng et al., 2017). Count data was imported to Seurat (version 4.0.4) for quality control (QC) filtering, clustering, dimensionality reduction, visualization, and differential gene expression (Satija et al., 2015; Hao et al., 2021). Each sample was filtered to cells containing at least 500 features with clusters requiring a minimum of 30 cells. Samples were merged prior to normalization and clustering (resolution 0.3). Differentially expressed genes between conditions for each cluster were identified using the “FindMarkers” function.

### Ingenuity Pathway Analysis

The functional pathways in each comparison were generated through the use of IPA (Qiagen Inc., https://www.qiagenbioinformatics.com/products/ingenuity-pathway-analysis). Genes with significant differential expression, as defined by a log fold change of ≥1 or ≤ −1, and a *p*-value<0.01, were uploaded into IPA software, using RefSeq IDs as identifiers.

### Apoptosis Assay

Immunohistochemistry was performed by the Wayne State University Biobank and Correlative Sciences Core. Formalin-fixed paraffin-embedded sections of bisected zebrafish were de-waxed and rehydrated in a xylene-ethanol-water series. Endogenous peroxides were removed by a methanol/1.2% hydrogen peroxide incubation at room temperature for 25 min. HIER antigen retrieval was done with a pH6 citrate buffer and the BIOCARE Decloaking Chamber (Concord, CA, United States). A 40 min blocking step with SuperBlock Blocking Buffer (Thermo Scientific, Waltham, MA, United States) was performed prior to adding the primary antibody. Detection was obtained using GBI Labs (Bothell, WA, United States) DAB Chromogen Kit and counterstained with Mayer’s hematoxylin. Sections were then dehydrated through a series of ethanol to xylene washes and coverslipped with Permount (Fisher Scientific, Waltham, MA, United States). A 1:100 dilution of Cleaved caspase three antibody (9664S) antibody (Cell Signaling, Danvers, MA, United States) was used overnight at 4 °C.

The authors analyzed CC3 labeling to determine presence and/or extent of apoptosis (control fish: *N* = 3; TCDD-exposed: *N* = 3). We obtained up to three distinct images from replicate testes slides at ×40 magnification, and manually quantified a quadrant of each image (control = 8 quadrants, TCDD = 9 quadrants). Significance of the percent apoptotic cells per cell type between controls and TCDD images was measured *via* student’s two-tailed *t*-test.

## Data Availability

The datasets presented in this study can be found in online repositories. The names of the repository/repositories and accession number(s) can be found below: NCBI GEO (GSE193758).
